# Supplementary material for: Prevalence of Different Salmonella enterica Subspecies and Serotypes in Wild Carnivores in Emilia-Romagna Region, Italy
Source: Animals (Basel). 2022 Nov 30;12(23):3368. doi: 10.3390/ani12233368 (PMC9738870; doi:10.3390/ani12233368)
Supplement: Supplementary file 1 [file animals-12-03368-s001.zip › animals-2056750-supplementary.pdf]

Supplementary Materials

Foxes

Study	Year	Country	Positive	Tested	Prevalence	95% CI
Study-2	1990	UK	3	188	0.02	[0.00, 0.04]
Study-5	2004	Spain	1	14	0.10	[0.00, 0.25]
Study-7	2006	Italy	19	462	0.04	[0.02, 0.06]
Study-10	2008	Norway	14	215	0.07	[0.04, 0.10]
Study-11	2013	Italy	63	1122	0.06	[0.04, 0.07]
Study-13	2014	Italy	29	509	0.06	[0.04, 0.08]
Study-15	2016	Poland	9	286	0.03	[0.01, 0.05]
Study-16	2016	Italy	22	261	0.09	[0.05, 0.12]
Study-17	2017	Austria	9	437	0.02	[0.01, 0.04]
Study-18	2021	Italy	17	200	0.09	[0.05, 0.13]
**Our Study**	**2022**	**Italy**	**42**	**718**	**0.06**	**[0.04, 0.08]**

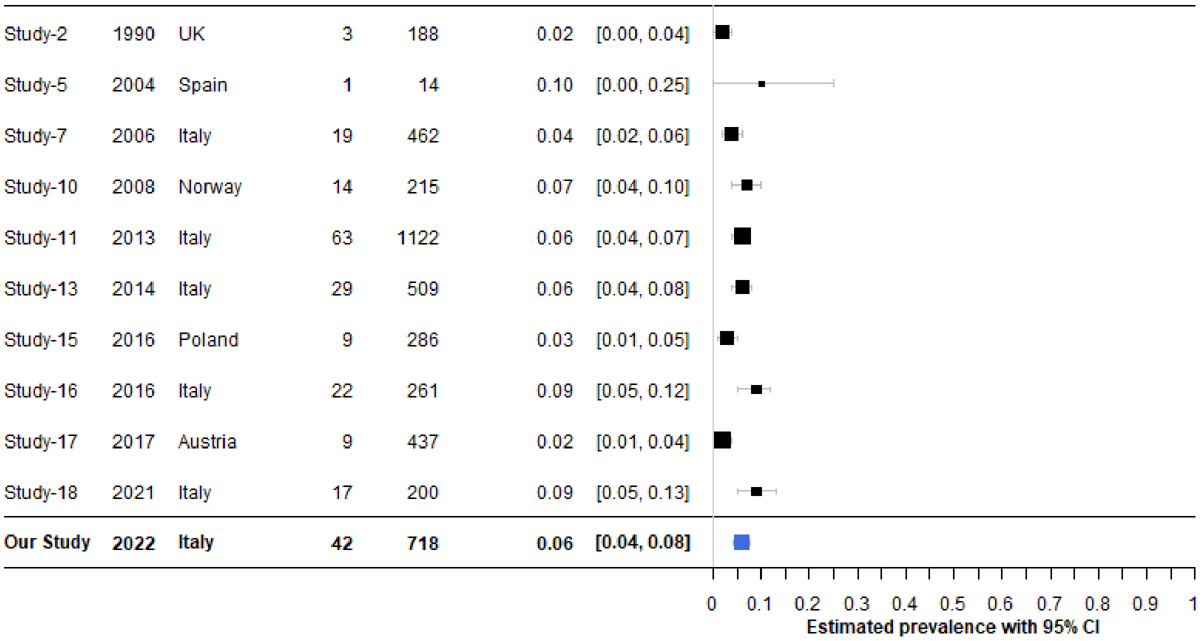

Figure S1. Number of the tested/positive foxes (*Vulpes vulpes)* from various European studies related to Salmonella prevalence, and respective Bayesian posterior estimated prevalence with 95%Credibility Interval. Study-2: Euden et al., 1990 [40]. Study-5: Millàn et al., 2004 [45]. Study-7: Remonti et al., 2006 [34]. Study-10: Handeland et al., 2008 [42]. Study-11: Botti et al., 2013 [13]. Study-13: Chiari et al., 2014 [12]. Study-15: Nowakiewicz et al., 2016 [37]. Study-16: Rubini et al., 2016 [36]. Study-17: Glawischnig et al., 2017 [28]. Study-18: Guidetti et al., 2021 [35].

Badgers

Study	Year	Country	Positive	Tested	Prevalence	95% CI
Study-1	1977	UK	49	585	0.08	[0.06, 0.11]
Study-3	1990	UK	350	4881	0.07	[0.06, 0.08]
Study-4	2003	UK	36	130	0.28	[0.20, 0.36]
Study-6	2004	Spain	4	22	0.20	[0.05, 0.36]
Study-8	2006	Italy	10	109	0.10	[0.04, 0.15]
Study-12	2013	Italy	25	221	0.11	[0.07, 0.16]
Study-14	2014	Italy	2	17	0.14	[0.01, 0.29]
Study-19	2021	Italy	17	85	0.20	[0.12, 0.29]
Study-20	2021	Northern Ireland	14	146	0.10	[0.05, 0.15]
**Our Study**	**2022**	**Italy**	**21**	**182**	**0.12**	**[0.07, 0.16]**

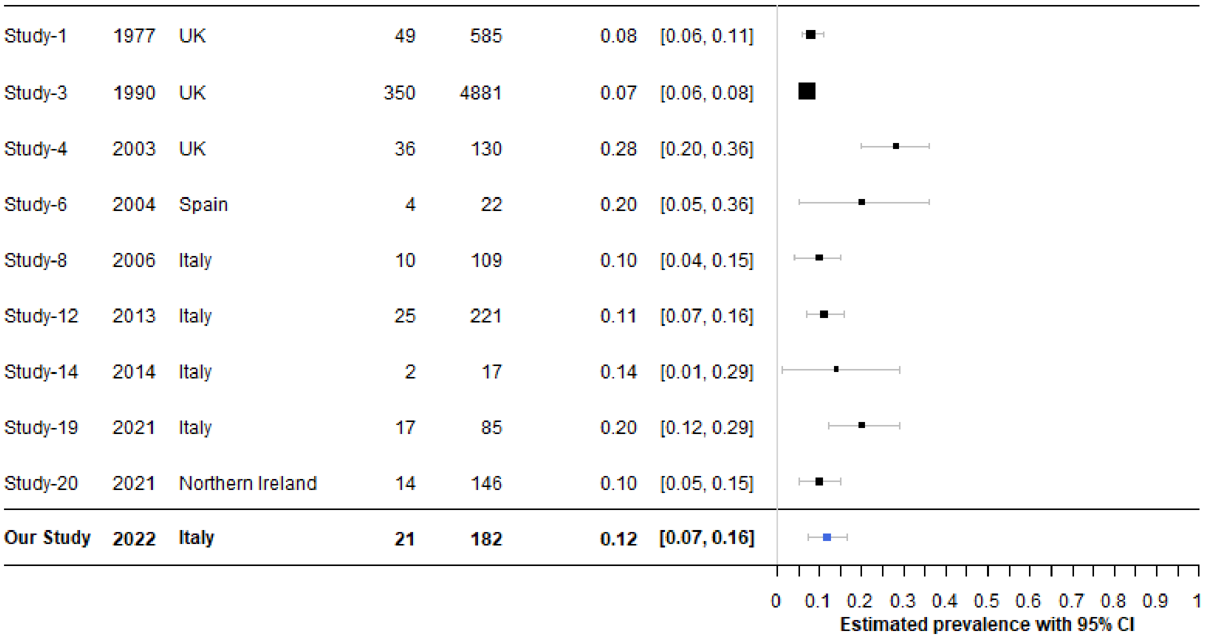

Figure S2. Number of the tested/positive European badgers (*Meles meles)* from various European studies related to Salmonella prevalence, and respective Bayesian posterior estimated prevalence with 95%Credibility Interval. Study-1: Wray et al., 1977 [47]. Study-3: Euden et al., 1990 [41]. Study-4: Wilson et al., 2003 [48]. Study-6: Millàn et al., 2004 [45]. Study-8: Remonti et al., 2006 [34]. Study-12: Botti et al., 2013 [13]. Study-14: Chiari et al., 2014 [12]. Study-19: Guidetti et al., 2021 [35]. Study-20: O'Hagan et al., 2021 [46].
